# Semimetal/Substrate Cavities Enabling Industrial Materials for Structural Coloring

**DOI:** 10.1021/acsaom.4c00521

**Published:** 2025-02-22

**Authors:** Fernando Chacon-Sanchez, Rosalia Serna

**Affiliations:** Laser Processing Group, Instituto de Óptica, IO-CSIC, Serrano 121, Madrid 28006, Spain

**Keywords:** structural color, alternative materials, ultrathin films, photonic cavities, bismuth, semimetals

## Abstract

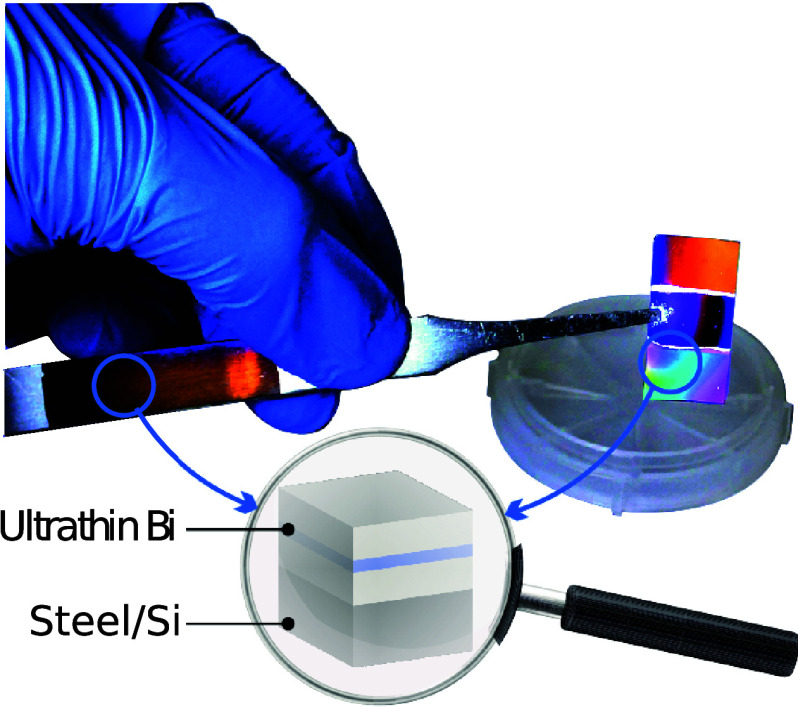

Color coatings are essential for the identification and safety of everyday objects as well as for the protection of surfaces from deterioration, in addition to their well-known use for enhancing their aesthetic appeal. However, conventional dyes and pigments are a major source of contamination and degrade easily over time. Structural coloring is a sustainable alternative capable of producing high-quality colors with nanometric structures. Nonetheless, many approaches to structural coloring rely on lithography or expensive back-reflectors made from noble metals. In this study, we approach surface coloring using lightweight, sustainable, and scalable optical coatings with subwavelength thickness. This method allows industrial surfaces to function as active elements in the color-generating structure, eliminating the need for metallic mirrors. The design is based on a semimetal/substrate cavity (SSC), directly deposited onto the surface to be colored. As a proof of concept, we designed and fabricated SSCs on silicon and stainless steel substrates, using ultrathin films of bismuth (Bi) and aluminum oxide (Al_2_O_3_) as the cavity components. These SSCs display vivid, well-defined colors with excellent angular stability for a cavity. Moreover, the SSC design can be adapted with other semimetal/dielectric combinations and offers an efficient, daylight-friendly, sustainable, and lightweight solution for functional coloration of everyday objects as well as components for industrial and technical applications.

## 1 Introduction

Structural coloring is based on the interaction of light with micro- and nanoscopic structures, and it is the main alternative to dyes and pigments regarding coloration, either in nature or in industrial applications. In nature, structural colors are ubiquitous, as almost every blue hue in animal appearance comes from structural coloring, and it can even be found in some plant tissues.^1^ However, their presence in manmade technology is still testimonial. One of the main reasons up to this day is the difficulty to scale up the processes to coat large surface areas, and therefore, they are not competitive over pigments and dyes. Nevertheless, many of the currently used pigments and dyes are pollutants, they are usually applied in nonsustainable processes, and they eventually degrade and colors fade away. Moreover, when used for large areas, they can become quite heavy for certain applications (e.g., automotive and aerospace). In contrast, structural colors do not fade away with time, and by choosing suitable materials and production processes, they can be fully sustainable and a lightweight alternative for application on large structures.

When designing reflective structural colors using metasurfaces and cavities, two primary types of active layer materials are typically employed: noble metals and phase change chalcogenides. Noble metals, such as gold (Au) and silver (Ag), are widely used because their high free-electron density imparts excellent plasmonic and resonant properties. These materials can function either as the active layer^2,3^ or as a reflective back mirror.^4−10^ However, their high cost, limited availability, and the complexity of their fabrication processes often make them unsuitable for large-scale industrial deployment. In contrast, chalcogenide materials—such as GeSbTe and SbSe compounds—enable tunable reflective colors by exploiting the nonvolatile solid–solid phase transition.^11−16^ Despite this advantage, these materials generally exhibit complex stoichiometries that complicate the deposition process, possess limited cyclability, and may contain either toxic or pollutant elements (e.g., Te). Promising advances, however, are being made with the use of elemental materials such as antimony (Sb).^17^

A key component of almost any structural color device is the optically thick metallic back mirror that is typically included as the bottom layer to reflect most of the incident light, with the aim to maximize the interaction between incident light and the active layer. Therefore, this back mirror forms part of the structural color device, and in order to estimate the thickness and hence the weight of the full structure, this optically thick layer has to be taken into account. To enable real-world applications of such cavities, it is essential to explore alternatives to noble metal mirrors. Furthermore, the ideal scenario toward real-world applications would be to use the actual surface of the object as the back mirror, integrating it into the coloring structure. However, reports of structural colors generated using industrial materials like silicon and steel are surprisingly rare, and most of them still include the earlier mentioned noble metals.^18−21^

In this context, we will focus on the structural coloring of silicon and stainless steel (referred to as steel from now on) surfaces. We have selected steel because it is used in everyday objects as diverse as kitchen sinks, cutlery, scissors, jewelry (like watches), home and medical appliances, sporting goods, and computers. Modern construction can also greatly benefit since steel-based structures are ever-present, starting from traffic road signs and pipe racks to heavy industrial buildings, as well as bridges, towers, and railway structures. Finally, automotive, aircraft, and space industries can also benefit from this approach because they employ steel for structural support, frames, and fuselage. On the other hand, pure silicon is not as widespread as a surface in our everyday life as steel, but it is an extremely abundant material and has great importance for critical industries like electronics and photovoltaics. The deployment of structural coloring based on nanophotonic structures for these objects instead of paint pigments can bring great benefits such as the already mentioned sustainability and durability and additional desired properties such as a much lighter weight, due to the inherent use of small amounts of material in the nanostructures, and protection against corrosion, due to the use of dielectrics as part of the structure. Another sustainable and extended material, as well as inexpensive, is aluminum. However, given its high reflectance, the generation of coloring is straightforward, and hence it has been widely studied.^22−25^

**Figure 1:**
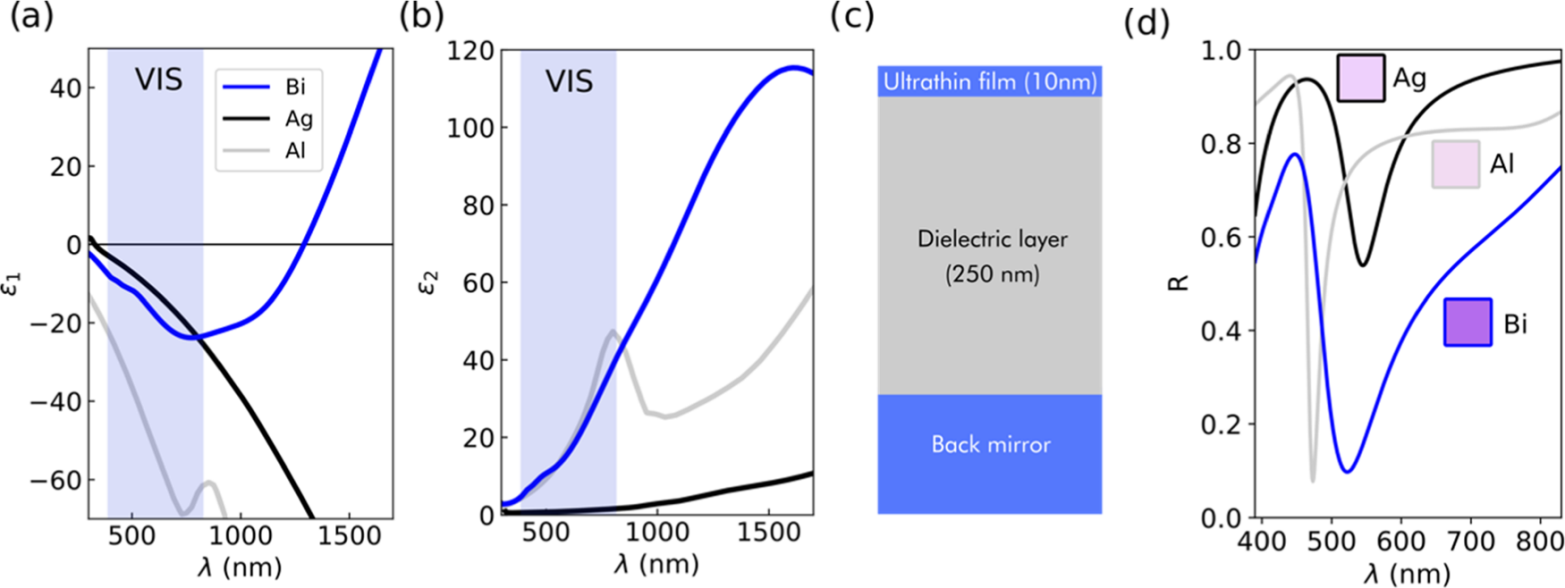
Material comparison of different MIM cavities. (a, b) Real and imaginary part of the dielectric function in the UV-NIR (300–1700 nm) range for selected commonly studied metals (Ag and Al) along with Bi. (c) General diagram of a MIM cavity . (d) Simulated reflectance curves in the VIS for MIM cavities with a-Al_2_O_3_ as the dielectric spacer and Ag, Al, and Bi as the thin film and back mirror materials (*t*_film_ = 10 nm, *t*_diel_ = 250 nm), with the obtained CIE colors as an inset.

Two of the most extended approaches to generate structural colors are nanostructured metasurfaces and Fabry–Perot-type cavities (FPC).^26^ Recently, in a previous publication,^13^ we have shown that vivid structural colors are successfully achieved by both approaches by implementing the semimetal bismuth (Bi) as the active material along with the usual metallic back mirror.^27^ While both metasurfaces and FPCs can be successfully used for structural coloring, the interference-based FPC is undoubtedly the most suitable approach for macroscopic coloring due to its easier scalability. Among the semimetals, we have chosen Bi because it is a sustainable,^28^ eco-friendly,^29^ single-element semimetal with unique properties that has been explored extensively on the field of condensed matter physics.^30−41^ However, it is only very recently that its use for nanophotonic applications has attracted attention.^28−34^ If we examine the dielectric function of Bi in Figure 1(a,b), we observe a lossy metallic-like behavior on the NIR-UV range, which makes Bi a suitable material for plasmonics,^42,43^ and thus Bi-based plasmonic metasurfaces have been fabricated.^27^ Bi is characterized by its high values of both real and imaginary parts of its dielectric function in the IR, caused by intense interband transitions. These interband transitions are the actual origin of its unique metallic-like behavior in the UV–vis spectral region that differentiates Bi from traditional metals that rely on free electrons.^44,45^ These giant values of the dielectric function mean that Bi has an extremely high refractive index in the IR (*n* ∼ 10), which can also be employed to design subwavelength metasurfaces.^46,47^ Finally, another remarkable property of Bi is its relatively low melting temperature (around 270 °C) and the significant differences in the optical properties between its solid and liquid states that enable the construction of reconfigurable photonic structures. Reconfigurable devices switchable either by conventional annealing^48^ or by fs-laser irradiation^49^ have been demonstrated.

In this work, we go one step further in the design and fabrication of photonic cavities with the aim of generating a palette of vivid structural colors for its implementation on everyday surfaces including industrial settings. For this purpose, we will design and fabricate semimetal/substrate cavities (SSCs), integrating in the structure the surface of typical industrial materials (Si and steel) and using the semimetal bismuth (Bi) in the structure as the active absorber. We will start our work with the demonstration that semimetals, in particular Bi, due to their unique dielectric optical properties show an enhanced performance for reflective structural color generation when compared to conventional metals when used in an ultrathin-film configuration.

## 2 Design Considerations

To reach the concept of semimetal/substrate cavities for industrial surfaces and sustainable, structural coloring, we have started by analyzing the response of archetypical FPC structures. The working principle behind FPC, including cavities based on metal–insulator pairs (MIM, MIMI) is optical interference, which is usually achieved by incorporating λ/4 dielectric layers between metallic films to generate destructive interference at the desired wavelength. In order to evaluate the importance of the choice of material for the active absorber film, we have performed simulations of an MIM (metal–insulator–metal) cavity for three different materials, a near-perfect electric conductor (silver, Ag), a lossy metal (aluminum, Al) and a semimetal (bismuth, Bi). Analyzing their dielectric function in the UV–vis–NIR range (λ between 300 and 1700 nm) in Figure 1(a,b), it is observed how Al reaches highly negative values for the real part of the dielectric function, whereas Ag and Bi show lower and similar values in the VIS. Regarding the imaginary part, associated with optical losses, it is observed how Ag shows very low losses, whereas Al and Bi show similar values with high losses in the VIS.

These qualitative similarities in the dielectric function are interesting; however, a more quantitative and direct method to compare the optical performance of these three materials is via simulations on a MIM cavity. Simulations were performed for the structure shown in Figure 1(c), with amorphous aluminum oxide (a-Al_2_O_3_) as the spacer dielectric layer with a thickness of 250 nm, and an active upper layer with a 10 nm thickness, in order to achieve an effective cavity with a reduced thickness. The back mirror is assumed optically thick, and for simplicity, we have implemented the same material (Bi, Ag, or Al) for both the back mirror and the top thin film of the cavity. The resulting reflectance has been simulated using a code based on transfer matrix method (TMM), and the result as a function of the wavelength is displayed in Figure 1(d). For the Ag cavity, we obtain a wide destructive interference, which is a desirable trait when generating pure reflective colors, but it is not intense enough to generate a vivid color, and the result is quite pale (see the inset of Figure 1(d)). On the other hand, for the Al cavity, a very intense destructive interference is generated, which is also a desired feature, but it has a very high *Q*-factor, and hence the generated color still lacks intensity and vivacity. In contrast, when Bi is used as the active absorber material, the resulting destructive interference is very intense and wide, and as a result, an intense and vivid color is generated (see the inset of Figure 1(c)). From these results, we can conclude that low negative values of the real part of the dielectric function ϵ_1_ generate wider destructive interferences, as seen for the case of the Ag-based cavity. And high values of the imaginary part ϵ_2_ such as those employed for the Al-based cavity generate more intense resonances close to near-perfect absorption for a 10 nm thick metallic film. In the VIS region of the spectra, the Bi-based cavity combines both aspects, with an ϵ_1_ very similar to that of the Ag, whereas the ϵ_2_ is very similar to the one of Al, resulting in a reflectance spectrum featuring both a wide and intense destructive interference that is ideal to generate reflective structural colors.

Finally, we would like to stress that, although for our purpose the choice of Bi is excellent, there are other semimetals (e.g., antimony, tin) and transition metals (e.g., nickel, titanium) with similar optical properties (i.e low but negative values of ϵ_1_ and moderate to high values of ϵ_2_) and, as it can be deduced from the above discussion, they would also be adequate materials.^7^ The design process would be identical to the one explained in this work.

Note that if higher orders are considered, then materials like Ag and Al can start showing better results, with higher maximum values of reflectance and sharper, purer colors (Figure S1 Supporting Information). The disadvantages of such a process will be the increase of the total cavity thickness, loss of angular robustness, and again, the use of nonsustainable and expensive materials.

Regarding the dielectric part of the cavity, we have selected aluminum oxide (Al_2_O_3_), which is an affordable sustainable material that is thermally stable and corrosion-resistant. However, its refractive index is comparatively low in the VIS range (ranges from 1.69 for λ = 390 nm to 1.66 for λ = 830 nm). The choice of a dielectric with a higher refractive index would improve the angular robustness of our structure, but given the mechanical properties, sustainability, and ease of fabrication, aluminum oxide stands as a compromise between maximum performance and real-world applicability.

Once we have established bismuth as an excellent choice to generate structural colors with an FPC-type structure, we will discuss how to improve the cavity design for effective and efficient structural color generation. While the simple MIM cavity shown in Figure 1 offers one of the most simple and robust approaches, it shows some disadvantages. The main limitation of this design would be that the upper ultrathin layer is directly exposed to the atmosphere. Therefore, it can be easily oxidized or corroded and its optical performance can be modified. This can be easily solved by adding a protective, thin dielectric layer on top of the Bi ultrathin layer, making it a metal–insulator–metal–insulator (MIMI) cavity that is fully suitable for outdoor use or in corrosive environments. Moreover, adding this dielectric layer shows the advantage of increasing the possibilities of the color design process given that in the MIMI configuration, the generated color is also a function of the thickness of the upper dielectric layer. From the technological point of view, the addition of an extra dielectric layer also contributes to simplify the production process because, as we will show, we can make designs in which the thickness of the underlying layers, i.e., the bottom dielectric and the ultrathin Bi layer, are fixed. In this type of design, the generated color is easily modified by just depositing the top dielectric layer with the desired thickness. Added to that, a specific benefit associated with the choice of Bi would be that the Bi layer could be active in response to thermal, electric, or optical stimuli, therefore allowing the development of optically tunable structures by exploiting the optical contrast related to its solid–liquid phase transition.^48,49^

As we discussed in the Section 1, toward the real-world application of these types of cavities, we must look for alternatives to expensive noble metal back mirrors. Establishing Bi as the ultrathin active material, we will compare the use as back mirrors of silver (Ag) as the archetypical near-perfect electrical conductor; aluminum (Al), as a more sustainable metallic alternative; and bismuth, as we have explained that it can be an excellent alternative material. And we will take our inquiry one step further and include materials that are not conventionally chosen as optical reflectors, such as silicon (Si) and steel. These materials are omnipresent in industrial settings, so integrating them directly as the back mirror could prove really advantageous to develop sustainable nanophotonic cavities for practical use on the surfaces of real, everyday objects.

**Figure 2:**
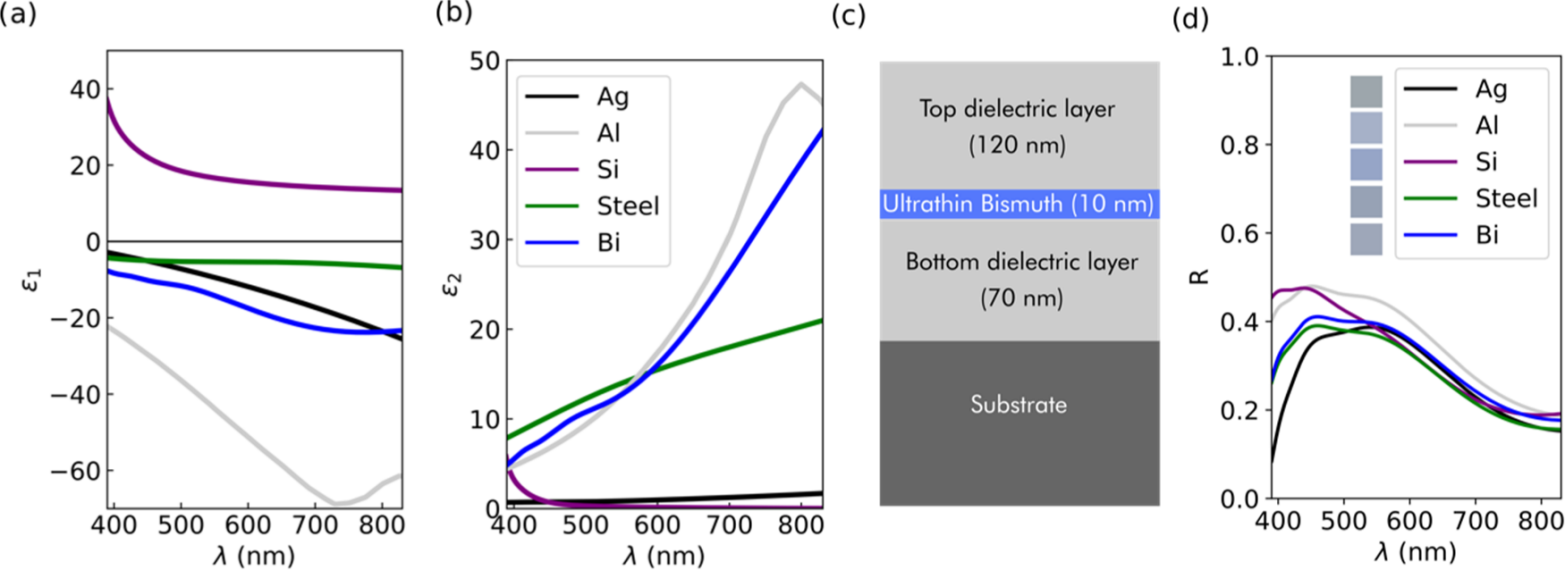
(a, b) Dielectric function in the VIS (390–830 nm) range for the selected materials Ag, Al, Bi, stainless steel, and Si, employed as the back mirror. (c) Diagram of the MIMI cavity. (d) Simulated reflectance curves for MIMI cavities with the structure shown in (c). The inset shows the obtained CIE colors. For the simulations in (d), Al_2_O_3_ has been selected as the dielectric, and Bi as the ultrathin active layer. The following layer thicknesses have been used: *t*_Bi_ = 10 nm, *t*_Al_2_O_3_,bottom_ = 70 nm, *t*_Al_2_O_3_,top_ = 120 nm.

Figure 2(a,b) shows the dielectric functions in the VIS spectral region for the selected materials for the back mirror of the structure. The Ag, Al, and Bi dielectric functions are shown and discussed previously in Figure 1(a,b). Steel behaves like a lossy metal, with relatively low absolute negative values of the real part ϵ_1_, along with significant losses with steadily increasing values of ϵ_2_ in the VIS. Note that although the ϵ_2_ values of steel show a moderate increase of a factor of 2, the corresponding ϵ_2_ values increase almost an order of magnitude in the same wavelength range for Al and Bi. Regarding Si, we can observe near-constant values of both ϵ_1_ and ϵ_2_, with always positive values for ϵ_1_, which is in agreement with its semiconductive nature in the studied spectral range. In order to compare the performance of these materials for structural coloring, we performed simulations of an MIMI cavity like the diagram in Figure 2(c). The designs integrated each material as the back reflector while keeping an ultrathin Bi film and a-Al_2_O_3_ as the spacer dielectric layer, with thicknesses of 70 and 120 nm for the lower and upper cavities, respectively. Although all of the dielectric functions are remarkably different between them, we can observe in Figure 2(d) that the resulting reflectance profile is very similar independently of the employed material for the substrate, with maximum values around 0.5 and similar resulting colors (bluish-gray). This assertion remains valid as long as we are operating within the constraints of the first interferential order, i.e., while maintaining for each dielectric cavity a maximum dielectric thickness , where *n*_diel_ is the refractive index of the dielectric (for Al_2_O_3_ between 1.66 and 1.69 in the VIS) and λ is the wavelength of the destructive interference (between 390 and 830 nm in the VIS). This condition is advantageous since this design requires small thicknesses, and we will see that the generation of a complete color palette is achieved.

In summary, considering this previous study, in the present work, we will investigate the structural color generation capabilities by the structure shown in Figure 2(c), with two sub-λ/4*n* dielectric cavities (bottom and top) and an ultrathin 10 nm thick semimetallic (Bi) layer in between. These SSCs will be deposited directly on industrially relevant materials, Si and steel. First, we will show how we have computationally designed and experimentally fabricated the subtractive primary color base of cyan, magenta, and yellow (CMY), with high purity and reduced angular dependence. Furthermore, in order to enable a practical and easy fabrication pathway, we have designed structures with only two different values for the thickness of the bottom dielectric layer and we have demonstrated how it is possible to generate a whole color palette that covers a great fraction of the sRGB color space by varying only the thickness of the top dielectric layer, within the first interferential order. This methodology allows us to obtain in a straightforward manner a rich palette of colors suitable for industrial deployment in large areas, compared to the use of a pixel combination that is necessary when only a CMY color base is generated.

## 3 Results and Discussion

### 3.1 Color Base Generation: Computational and Experimental Results

**Figure 3:**
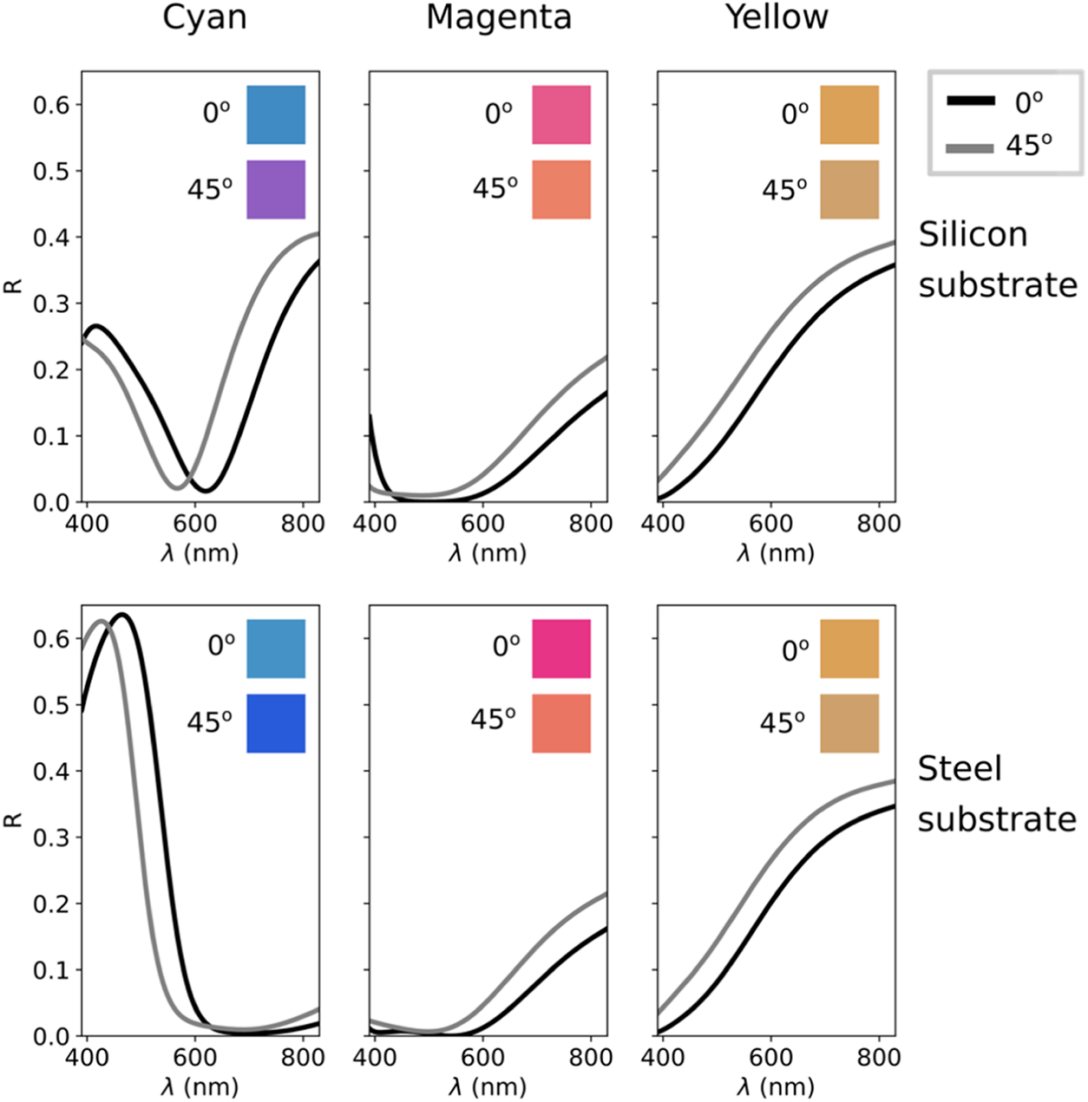
Computationally designing a CMY color base. Reflectance curves for the optimized CMY color base at normal and 45° incidence, for Si and steel as the substrate and Bi as the semimetal. As an inset in each curve, the obtained CIE colors at normal incidence and 45° are displayed.

Focusing on the structural color generation capabilities of our SSC design, we started by performing a computational analysis on the viability and color purity of our approach, with the material choices of Bi as the active absorber, Al_2_O_3_ as the dielectric, and Si and steel as the substrates. Here, as we have explained in Section 1, we will focus on reflective colors with the main objective to enable the coloring capabilities of silicon and steel surfaces. Although the thickness of the ultrathin bismuth film (Bi ut) could be a variable parameter, for the sake of simplicity, in this study we will focus on generating vivid, pure structural colors by modifying only the thickness of both dielectric layers. Adding a random search algorithm to our TMM-based code (see Section 5 for details), we first designed colors as similar to CMY as possible (at normal incidence), obtaining vivid, pure colors, as can be observed in the computational results displayed in Figure 3. Strong angular dependence on reflectance is generally regarded as a usual drawback associated with the performance of MIMI (or any FP-based) cavities; therefore, it is interesting to evaluate the angular performance of these SSCs. Once we had obtained the optimum values of dielectric thickness for each color, we additionally obtained the resulting reflectance (*R*) and CIE color at 45° of incidence. In Figure 3, we can see that they display greater angular robustness than similar interference-based coloring structures.^26^ However, there is still some non-negligible angular dependence on the reflected color with a blue shift of a few tens of nanometers when the angle of incidence increases from 0 to 45°. This slight blue shift is a consequence of the wide destructive interference and arises from the fact that most of the designed SSCs generate colors within the first interferential order, as we will discuss later in more detail.

Further analysis of Figure 3 reveals near-zero reflectance values at the minima of the spectra for the cyan and magenta colors. These observed low minimum values enhance the contrast and purity of the observed colors. Furthermore, the maximum reflectance values are moderate (0.2–0.6) which makes the SSCs suitable for potential daylight use, avoiding potential glaring issues toward real-world applications. If we compare the difference between the obtained colors for the two different used substrates, silicon and steel, we can appreciate very similar colors and reflectance profiles except for cyan. This difference can be readily explained by examining the obtained values for the optimized dielectric layer thicknesses. A more quantitative analysis of the color purity is continued in Section S3 in the Supporting Information.

**Figure 4:**
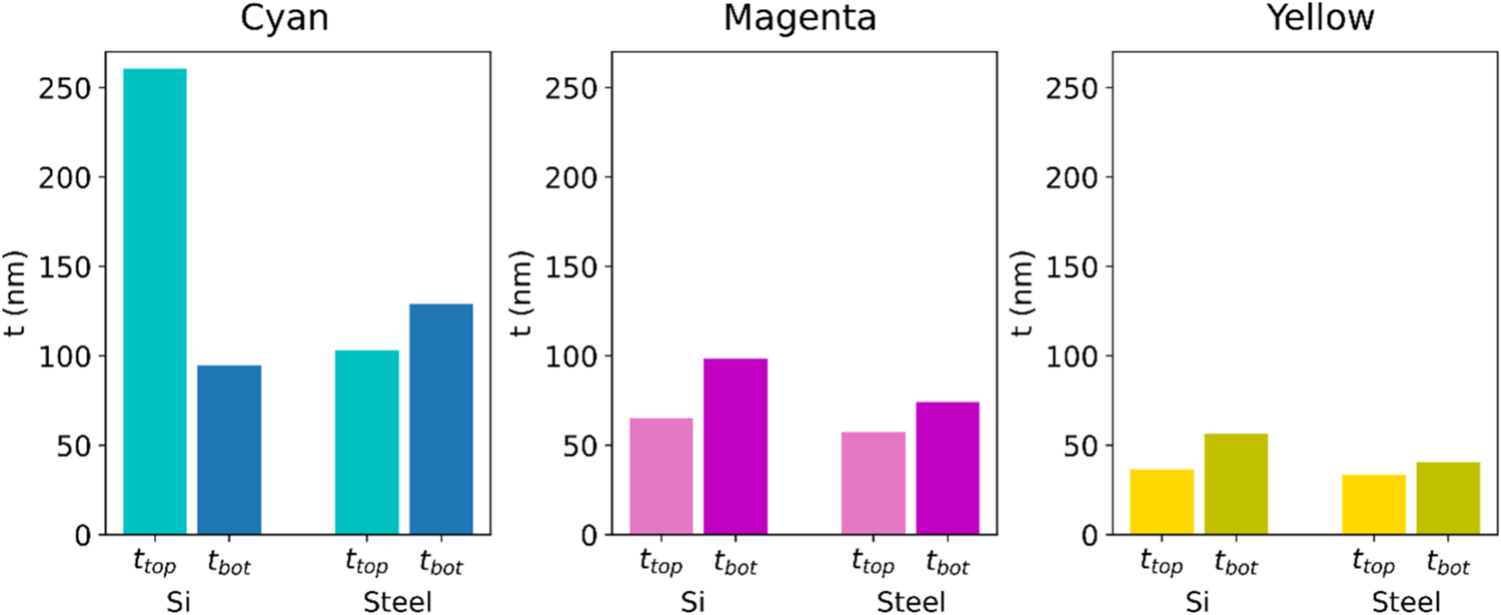
Computationally optimized dielectric (Al_2_O_3_) thickness for each dielectric layer to generate the CMY color base for Si- and steel Bi-based SSCs.

Figure 4 shows the computationally determined thickness of the dielectric Al_2_O_3_ layer for each color/substrate combination. Following the considerations taken in the discussion of Figure 2, we designed our SSCs so that their optical response is within the first interferential order. Given that the refractive index of Al_2_O_3_ in the VIS is almost constant (ranges from 1.69 for λ = 390 nm to 1.66 for λ = 830 nm), the maximum thickness of each cavity should be . We can observe in Figure 4 that this condition is fulfilled for all color/substrate possibilities except for the cyan/Si combination. For this specific color/substrate combination, it was necessary that the dielectric thickness was more than 2 times that for its steel equivalent. This is due to the low losses of Si toward the IR spectral region, along with the extremely high refractive index of Bi. The combination of these two factors hinders the generation of intense destructive interferences in the 600–800 nm range, so the design algorithm tends to take the second interferential order. For all of the other color/substrate combinations, we can observe that similar thickness values are needed to achieve similar colors for each Bi/Si and Bi/Steel SSC, even with a very similar proportion between the thickness of both top and bottom dielectric layers. Obtaining very similar SSC structures for materials with such different dielectric functions as silicon and steel (as it was hinted in Figure 2) highlights the versatility and robustness of our approach and suggests that the semimetal thin film layer plays a key role in the cavity structure.

As it was mentioned in Section 2, this approach could be employed by integrating different semimetals and transition metals as the active material, and the SSC can also be implemented in a myriad of different substrates. In Figure S5, we have added simulations of the CMY-obtained colors and total thicknesses for different active materials (antimony and titanium), also adding abundant and inexpensive aluminum as a potential substrate.

**Figure 5:**
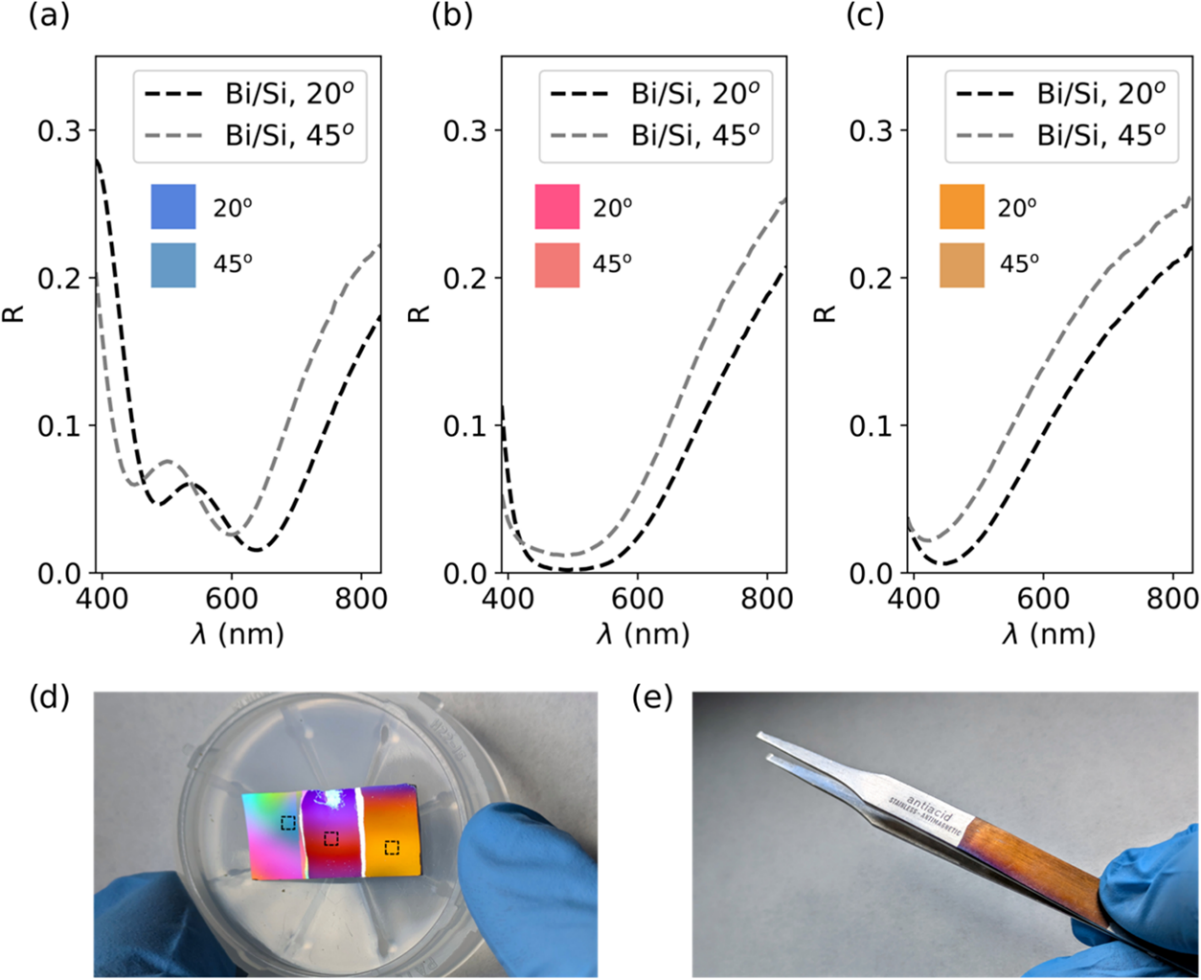
Experimental results. (a–c) Reflectance curves of the fabricated Bi/Si SSCs for the CMY color base: (a) cyan, (b) magenta, and (c) yellow. Resulting CIE colors are displayed as an inset for 20 and 45° of incidence. (d) Photography of the sample on Si under natural (solar) illumination, where we have performed gradient deposition, with the measured areas marked. (e) Photography of the Bi/steel SSC generating the yellow color, fabricated over a pair of standard stainless steel tweezers.

In order to experimentally validate the viability of the computational results, we have fabricated the three Si-based colors, which are shown in Figure 5. We chose to fabricate the Si-based colors since the cyan/Si is the combination that could prove more problematic to obtain since thicker dielectric layers are needed (Figure 4).

In Figure 5(a–c), we have included the reflectance measurements for 20 and 45° of incidence, along with each resulting CIE color. We can observe very similar results to those of the designed SSCs in Figure 3, with cyan being again the color with the most divergence. In this case, we can attribute the difference to potential deposition issues related to the comparatively high dielectric thickness. However, as can be observed in the insets of Figure 5, the measured colors are in very good agreement with the colors obtained from the simulations in Figure 3. Detailed results on the characterization through ellipsometry of the fabricated cavities can be found in Section S4 in the Supporting Information.

Regarding the fabrication process, employing PLD we performed a graded deposition of the dielectric. As a result, we generated a set of colors around each component of the color base and thus explored additional color generation capabilities for the SSCs. The resulting colors are displayed in Figure 5(d), with markers on each measured area. Additionally, in order to prove that our approach is indeed valid for real-life objects and surfaces, we performed the same gradient deposition employed for the yellow color in a pair of standard stainless steel tweezers, obtaining large area and satisfactory results, which can be observed in Figure 5(e).

### 3.2 Full Color Palette Generation

Once we have successfully demonstrated both computationally and experimentally the realization of a pure, daylight-friendly CMY color base, we have aimed to achieve the generation of a full color palette with an efficient approach. This objective is justified because even if theoretically by mixing microscopically pixel-sized elements of each element of our color base it is possible to obtain every desired color, the experimental process can prove to be very costly and inefficient, and hence it is not suitable for industrial deployment. In order to streamline the adaptation process of our SSCs to real-world applications, it would be desirable to generate each individual color directly from a single structure, therefore minimizing the fabrication process steps required to achieve the different colors.

**Figure 6:**
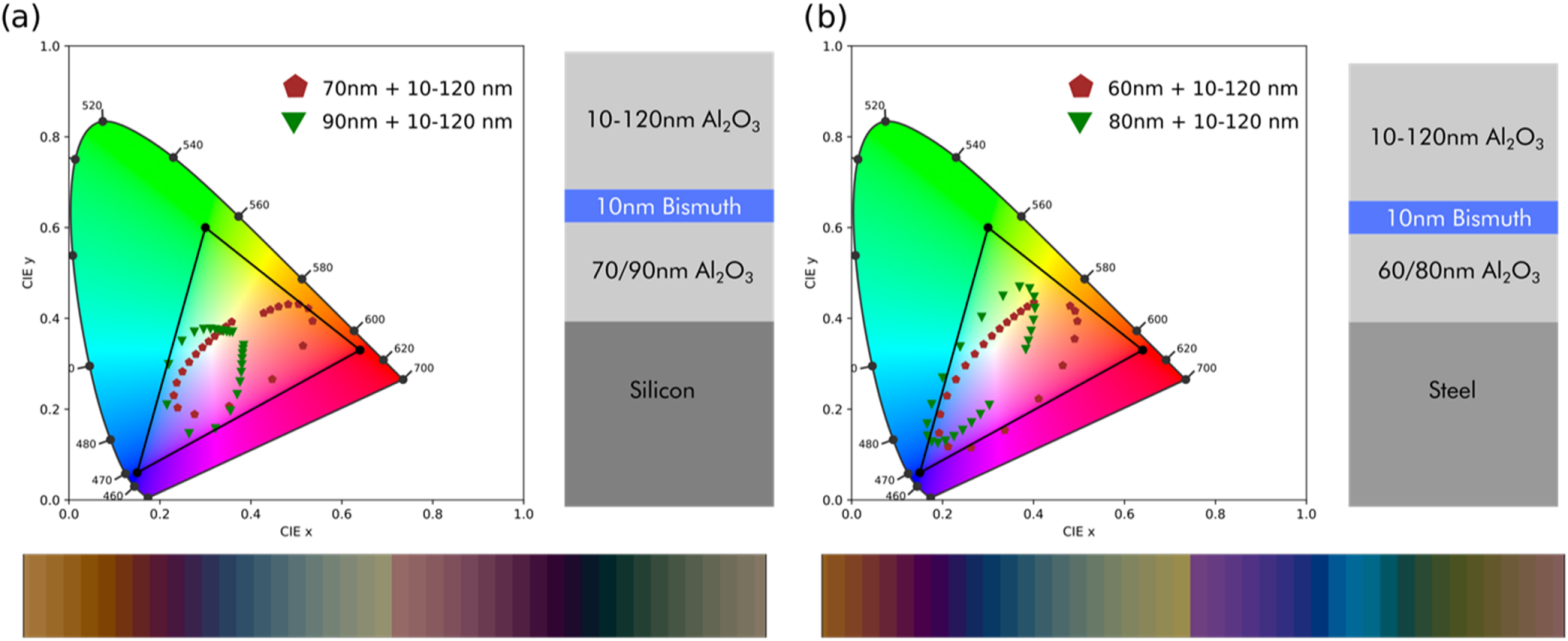
Near-sRGB generation and computational results. (a) CIE coordinates of the two used structures for Bi/Si substrate SSC, with each point being the resulting color of a structure with ±5 nm thickness of *t*_top_, along with a schematic representation of the corresponding SSCs on the right and the generated RGB color palette at the bottom. (b) CIE coordinates of the two used structures for Bi/steel substrate SSC, with each point being the resulting color of a structure with ±5 nm thickness of *t*_top_, along with a schematic representation of the SSCs on the right and the generated RGB color palette at the bottom.

With that objective in mind, we explored robust configurations for our SSCs in order to reduce the free design parameters. We found that a very efficient approach is to have a fixed value for both the thickness of the bottom dielectric and Bi layers (*t*_bot_ and *t*_Bi_) and obtain an entire color space just changing the top dielectric film thickness (*t*_top_). We have focused on the sRGB color space since it is the most extended for printing and display applications. Employing the previous TMM-based code, we successfully designed a complete color palette, as it can be observed in the computational results in Figure 6. On each CIE diagram, we can see the resulting CIE coordinates (colors) of cavities with a fixed *t*_bot_ and *t*_Bi_ and a variable *t*_top_ between 10 and 120 nm, i.e., within the first interferential order. In the sRGB color space, the purer colors are the ones located on the edges of the RGB-formed triangle, so our objective would be to obtain as many points near the edge as possible. With just 2 fixed configurations for *t*_bot_ and a fixed *t*_Bi_, we managed to cover a large fraction of the edges of the RGB triangle (i.e., a great number of pure colors), for both Si and steel as substrates. The obtained colors can be seen in the lower part of Figure 6(a,b).

## 4 Conclusions

In the midst of the urgent need for comprehensive industrial remodeling toward sustainability, structural colors emerge as a promising viable alternative to traditional dyes and pigments. Toward real-world applications for everyday objects, a thorough assessment of the chosen materials and approach is critical. We focused this study on alternatives to overcome the shortcomings of the most extended approaches. Hence, we propose a minimalist approach to create novel structural color optical coatings, formed by ultrathin layers of sustainable materials, Bi and Al_2_O_3_, and without a dedicated back mirror. In particular, we show that MIM cavities based on ultrathin semimetal films can show a better performance than those built by using archetypical metals and generate vivid and pure colors. Furthermore, we show that these structural colors can be efficiently produced integrating widely extended material surfaces such as silicon and stainless steel in the structure, creating the semimetal/substrate cavities (SSCs) that eliminate the need of a dedicated metallic back mirror. We demonstrate the suitability of bismuth as an excellent material to explore this type of structures. Based on this SSC approach, we have successfully designed and fabricated first a CMY color base, with pure, daylight-friendly colors as a proof of concept. In addition, we computationally demonstrate the feasibility of the generation of a color space similar to the standard sRGB color space while implementing a straightforward methodology that suggests a practical starting point for industrial process implementation. Finally, it is especially remarkable that an excellent SSC coating performance has been successfully demonstrated for two very different industrial substrates: silicon and stainless steel. From these results, we resolve that the approach to structural color through SSC is extremely versatile, opening a promising venue to integrate other surfaces for coloring and to test the performance of different active materials within the cavity. Following this approach, the surface structural coloring proposed approach could be suitable for everyday objects, including those that require large areas like construction, automotive, aircraft, and space industries.

In conclusion, we have provided a successful methodology for the development of ultralight, cost-effective optical coatings based on alternative sustainable materials, highlighting that conventionally employed materials in academic studies may not always be a unique choice and emphasizing the importance of conducting a thorough assessment of the selected material beforehand.

## 5 Materials and Methods

### 5.1 Experimental Fabrication and Characterization

Thin film deposition: Deposition of Bi and Al_2_O_3_ was performed by pulsed laser deposition (PLD).

In order to achieve a gradient deposition via PLD, the substrate was displaced by a few centimeters from the center of the plasma plume where a maximum deposit is achieved. Details about the morphology and optical properties of the employed ultrathin Bi films are explained in more detail in Section S2 in the Supporting Information. For the steel and Si substrates, we have used commercially available materials. For Si, we have used (100) Si wafers. For the stainless steel simulations, we characterized steel 1.7131 (16MnCr5), mirror-polished down to a root-mean-square roughness RRMS < 20 nm. We have also successfully tested the deposit on stainless steel tweezers.

The dielectric functions and the deposition rates of all of the materials employed in this work, either as substrates or as thin films (namely, Si, steel, Al_2_O_3_, and ultrathin Bi), were previously characterized by spectroscopic ellipsometry (Woolam VASE, spectral range 250 nm to 1770 nm). The deposition rates were confirmed via atomic force microscopy. Note that this characterization is especially important for thin films because their optical properties might vary significantly depending on the deposition conditions of the selected materials. Reflectance of the SSCs was measured for different angles of incidence over small areas (∼0.3 mm^2^). This allowed us to determine with accuracy the areas with thickness gradients observed in Figure 5(d), measuring in an effectively uniform area. In combination with the reflectance measurements, we also performed an ellipsometric analysis on the SSCs, and from this analysis, we can univocally obtain the deposited thicknesses on the measured area. More details about the deposition are included in Section S5 in the Supporting Information.

### 5.2 Computational Design and Analysis

Once the dielectric function of each employed material was determined, they were used for the modeling and optimization of the SSC response. The simulations were carried out employing a transfer matrix method (TMM)-based Python code, based on the procedures explained in ref (50), with the addition of the basic color optimization algorithm.^50^

In order to find the optimum dielectric thickness, a simple random search algorithm was designed and implemented. This following iterative procedure was implemented: First, we started the algorithm with initial conditions for the thickness of both dielectric layers within the first interferential order , calculating the obtained CIE coordinates according to the previous section. Macroscopic color calculations were carried out using Python’s Color library to obtain colors from theoretical and experimental reflectance spectra using the standard CIE1931 color space. Then, we made a random, small displacement in both thicknesses (taken from a Gaussian distribution centered in 0 with a standard deviation of 1 nm) and calculated its corresponding macroscopic color. If the obtained CIE coordinates were closer to the objective (namely, cyan, magenta, yellow) than the one we previously had, then the new thicknesses were accepted as our new conditions. This process is iterated until we reached a stationary solution with satisfactory results, minimizing the modulus of the distance in the CIE coordinates. Different initial conditions were explored in order to make sure the solution was unique and optimum within reduced dielectric thicknesses. This process was repeated for each CMY color and theoretically could be performed for any desired CIE coordinates.

## Data Availability

Data will be shared upon reasonable request to the corresponding authors.

## Supporting Information Available

Simulations of MIMI cavities for higher interference orders (Section S1); characterization details about the ultrathin Bi films (Section S2); additional considerations about the material choice and simulations of the CMY color base generation for different material combinations (Section S3); characterization details about the fabricated Bi/Si CMY color base (Section S4); and additional details of the deposition process (Section S5) (PDF)
