# Supplementary material for: Semimetal/Substrate Cavities Enabling Industrial Materials for Structural Coloring
Source: ACS Appl Opt Mater. 2025 Feb 22;3(3):727–36. doi: 10.1021/acsaom.4c00521 (PMC11959862; doi:10.1021/acsaom.4c00521)
Supplement: Supplementary file 1 — ot4c00521_si_001.pdf [file ot4c00521_si_001.pdf]

Supporting information for:

Semimetal/Substrate Cavities Enabling Industrial Materials for Structural Coloring

Fernando Chacon-Sanchez* and Rosalia Serna
*fernando.chacon@csic.es
Laser Processing Group, Instituto de Óptica, IO-CSIC, Serrano 121, Madrid 28006 Spain

S1. Reflectance and generated colors for higher interference orders in MIMI cavities

In order to clarify the role of the substrate on the resulting colors of the MIMI cavities generated for higher interference orders we have simulated cavities with a variable thickness in the VIS range. Figure S1a) shows the reflectance for cavities with different substrates working as a back reflector and the obtained color. Figure S1b) shows the corresponding diagram of the cavity with the thickness values that have been used. For the study, in order to illustrate the effect of higher orders, we have selected both thicknesses values above the $t = \lambda/4n$ threshold that denotes the first interferential order. The resulting CIE colors are distinct for each substrate and are more intense and vivid for the more metallic materials, in contrast with the results on Fig. 2 on the paper. Note that the thickness values used for the higher orders are considerably larger, more than factor of 2, than those used in Fig 2.

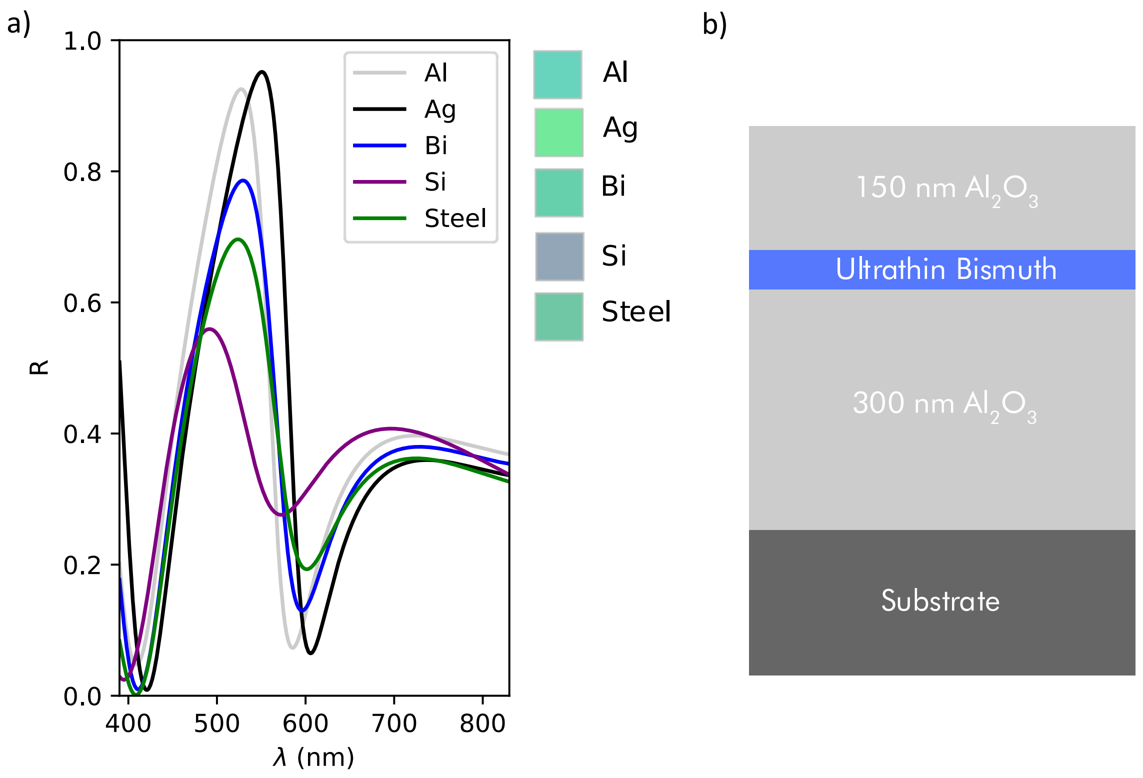

Fig. S1. a) Simulated reflectance and the resulting CIE colors for the cavity diagram shown in b) for 5 different substrates used as backrelfector (Al,Ag, Bi, Si, Steel)

S2. Characterization of the Bi ultrathin films.

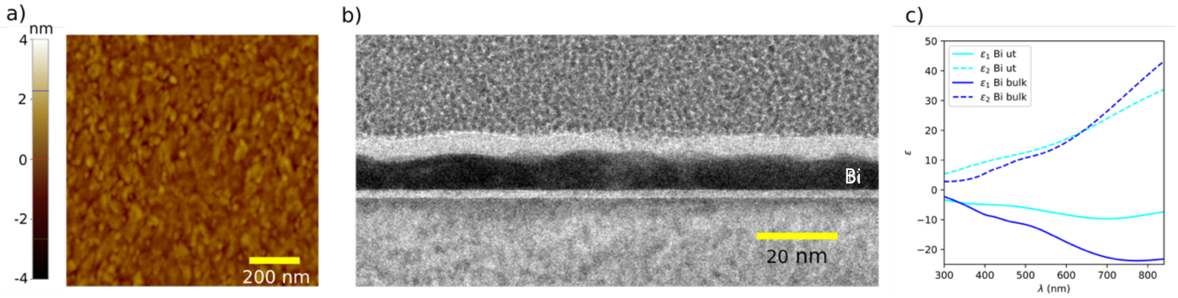

Fig. S2. Characterization of high-quality ultrathin Bi films. a): AFM image of the surface of a deposited ultrathin Bi film. They are polycrystalline, with a very smooth surface and a measured mean square roughness of 0.47nm. b): TEM cross-section image of the deposited Bi films, grown directly over a Si (+2nm SiO_2) substrate, and below a Al_2O_3 coating. We can observe that the film thickness is clearly in the 10 nm range, with a roughness in the nanometer range, similar to AFM measurements. c) Dielectric function of the deposited thin films and the bulk material in the VIS range.

Historically, fabrication and characterization of the optical response of Bi films has proven to be challenging. A comprehensive study of the optical properties of bulk Bi films (thickness > 100 nm) with a measured dielectric function covering from the ultraviolet to the near infrared was not published until the last decade. [1] When reviewing the values reported for the dielectric function in the ultrathin regime, around 10 nm thick films, it is found that they diverge significantly from those of the bulk material.[2–4] This divergence with the bulk values has been attributed to surface roughness and oxidation.[4] Exploring different deposition parameters and fine-tuning the process we have successfully deposited smooth, high density, ultrathin Bi films with optical properties similar to the ones of the bulk material. To determine its optical properties, we have performed spectroscopic ellipsometry. The determined dielectric function is in very good agreement with the one of bulk Bi in the VIS range, as can be seen in Fig. S2 c). The slight differences between our ultrathin material and the bulk material could be attributed to electron scattering in the multiple grain boundaries between crystallites or even some finite-size effects [5], but a more detailed analysis, beyond the reach of this study, is needed to univocally discern its origin.

In order to fully characterize our ultrathin Bi films dielectric response, we performed spectroscopic ellipsometry (SE) on the films in the UV-NIR range (250-1770 nm). We modelled the imaginary part of the dielectric function (ε_2) of the ultrathin Bi films via Lorentz oscillators. Whereas in [6] characterization of Bi is done using up to 9 oscillators, we simplified the model and used only 4 oscillators, with their parameters on Table S1, with a resulting thickness of 11.6 nm and a value of the Mean Square Error (MSE) of 2.342. *A* designates the amplitude of the Lorentzian oscillator, *En* designates the center of the oscillator and *Br* denotes the width of the oscillators. After obtaining ε_2, ε_1 is obtained via Kramers-Kronig consistency. The resulting function for ε_2 is shown in Fig. S3.

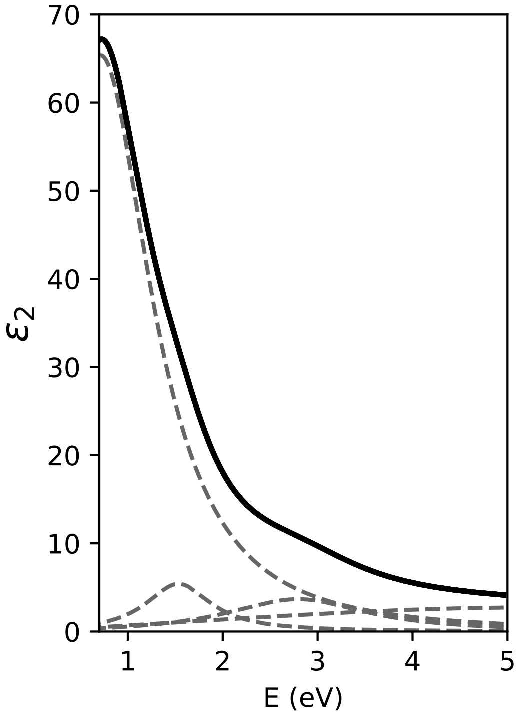

Oscillator nº	A	En (eV)	Br
1	56.86	0.96	1.40
2	3.59	2.93	1.80
3	2.40	7.03	10.0
4	5.32	1.59	0.84

Fig. S3. Resulting imaginary part of the dielectric function. Each component oscillator is shown in the table

S3. Analysis of the the CMY colorbase computational generation.

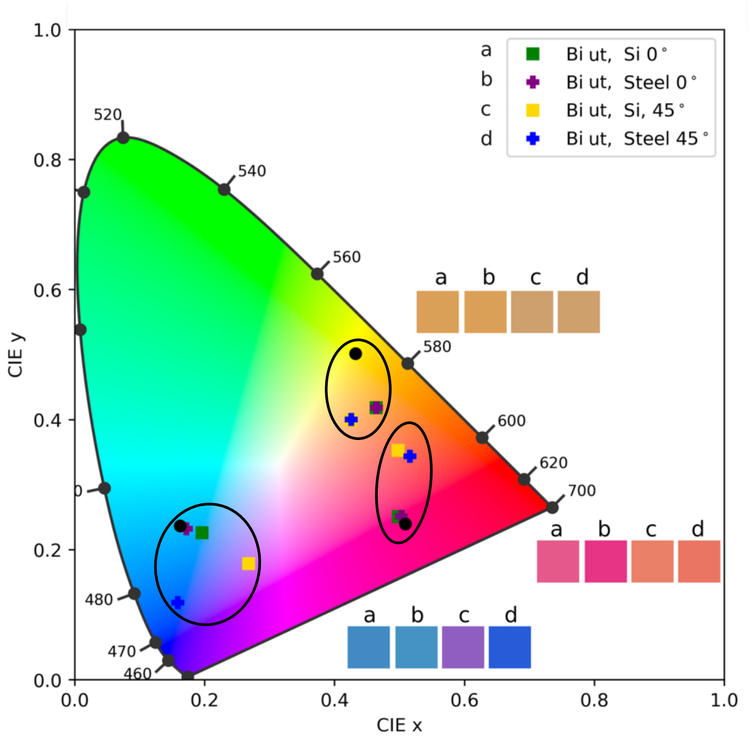

Fig. S4. CIE coordinates of the simulations for the CMY colorbase for Bi/Si and Bi/Steel SSC.

For a quantitative analysis on color purity, in Figure S4 we have plotted the CIE coordinates of the Bi/Si and Bi/Steel CMY color base, for normal and 45º incidence. The distance in the CIE diagram is employed as a metric to quantify the color purity of the SSC. It can be seen that normal incidence colors are closer to the target coordinates.

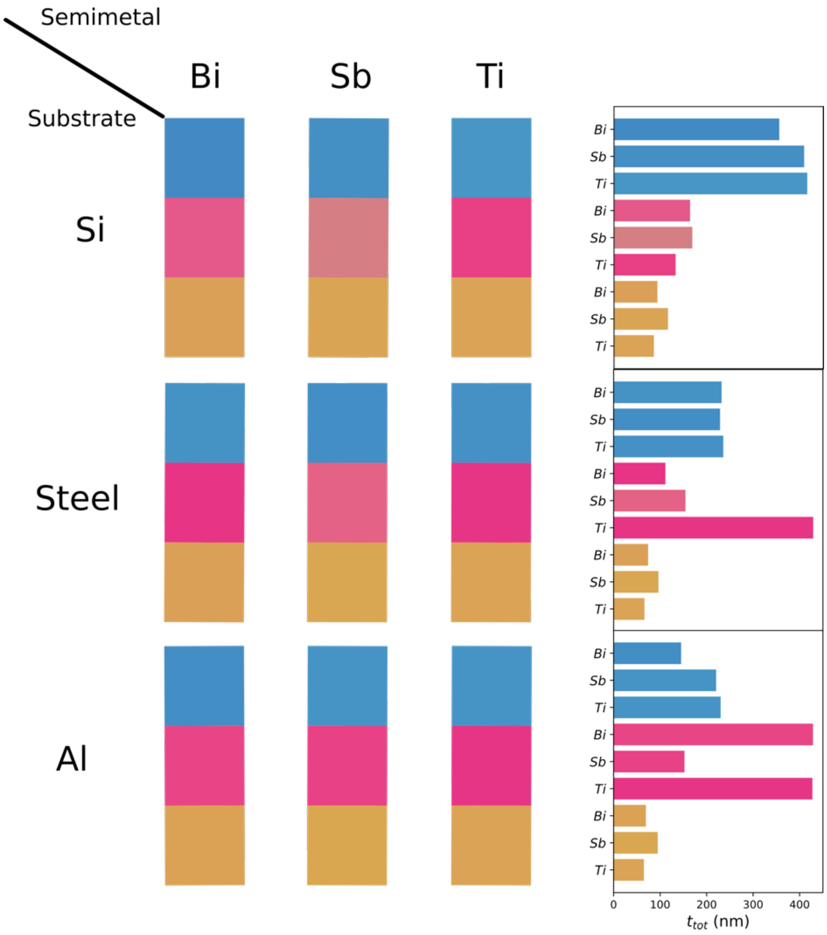

Fig. S5. CMY simulations for Bi, Sb, Ti active materials and Si, Steel and Al substrates, along with the total dielectric thicknesses for each color. Ti, Al and Sb optical properties are taken from [7] (Ti, Al) and [8] (Sb).

Additionally, as discussed in sec. 2 in the manuscript, this approach can be readily extended to other active materials besides Bi, and to other substrates besides Si and steel. In order to strengthen this affirmation, we have performed simulations for the CMY color palette with two other active materials, antimony and titanium, and to another sustainable and widely used substrate, aluminium. In figure S5 we can observe pure colors for each combination, and similar thicknesses for most of the cases. There are a few exceptions, the Si/cyan combination, that is discussed in the manuscript, and the Ti/steel/magenta and Bi,Ti/Al/magenta. The singular behavior of the two latter cases can be attributed to the combination of materials with a more metallic behaviour, that tend to generate sharper destructive interferences. Therefore, in those cases in order to obtain the broad absorbance required for magenta it is necessary to combine two destructive interferences to achieve a satisfactory result.

S4. Experimental details about Si-based CMY colorbase.

In addition to the reflectance measurements, we experimentally characterized the fabricated cavities for the Bi/Si CMY color base via ellipsometry and reflectance measurements. We compare in table S2 the thickness values experimentally obtained (exp) with those computationally obtained from the optimization algorithm (comp). The correspondence is good, with maximum divergences of a few nanometers.

	C_{exp}	C_{comp}	M_{exp}	M_{comp}	Y_{exp}	Y_{comp}
t_{top}(nm)	257.66	260.6	64.37	65.1	25.79	36.7
t_{Bi}(nm)	4.97	9.6	14.41	9.6	8.17	9.6
t_{bottom}(nm)	96.82	94.8	94.48	98.5	57.10	56.6
Substrate	Si	Si	Si	Si	Si	Si
MSE	21.68		5.77		5.28	

Table S2. Parameters obtained from the ellipsometric modellization of the CMY Si-based colorbase

S5. Additional details of film preparation via Pulsed Laser Deposition

The SSC structures developed in this work have been prepared by pulsed laser deposition (PLD). One of the main advantages of PLD compared with other deposition methods is that, since the evaporated species acquire a high kinetic energy (100-150 eV), the generation of high-quality dense material is straight forward and this optimization process is greatly reduced in time, and it is faster to obtain high quality ultrathin films. Furthermore, it has been shown [9] that employing PLD it is possible to fabricate homogeneous devices with areas up to cm^2. For the PLD thin films preparation in this work an ArF excimer laser (λ = 193 nm) was employed, with a pulse duration of 20 ns, and a repetition rate of 10 Hz and 20 Hz for Bi and Al_2O_3 respectively. The base pressure inside the vacuum chamber was in the range of 3 x 10^{-6} mbar, which was found to be optimum for Bi deposition. Additionally, for Bi deposition we added an optical attenuator to further reduce the energy of the laser reaching the Bi target. Deposition rates were 0.18nm/s for Bi and 0.04nm/s for Al_2O_3.

References

(1) Toudert, J.; Serna, R.; Camps, I.; Wojcik, J.; Mascher, P.; Rebollar, E.; Ezquerra, T. A. Unveiling the Far Infrared-to-Ultraviolet Optical Properties of Bismuth for Applications in Plasmonics and Nanophotonics. *Journal of Physical Chemistry C* **2017**, *121* (6), 3511–3521. https://doi.org/10.1021/acs.jpcc.6b10331.

(2) Hao, J.; Tan, C.; Zhou, D.; Qiu, Q.; Wen, Z.; Sun, Y.; Zhang, J.; Han, M.; Dai, N. Deep-Subwavelength Multilayered Meta-Coatings for Visible-Infrared Compatible Camouflage. *Nanophotonics* **2024**, *13* (13), 2391–2400. https://doi.org/10.1515/nanoph-2024-0029.

(3) Ghobadi, A.; Hajian, H.; Gokbayrak, M.; Butun, B.; Ozbay, E. Bismuth-Based Metamaterials: From Narrowband Reflective Color Filter to Extremely Broadband near Perfect Absorber. *Nanophotonics* **2019**, *8* (5), 823–832. https://doi.org/10.1515/nanoph-2018-0217.

(4) Toudert, J.; Serna, R.; Deeb, C.; Rebollar, E. Optical Properties of Bismuth Nanostructures towards the Ultrathin Film Regime. *Opt Mater Express* **2019**, *9* (7), 2924. https://doi.org/10.1364/ome.9.002924.

(5) Hirahara, T.; Nagao, T.; Matsuda, I.; Bihlmayer, G.; Chulkov, E. V.; Koroteev, Y. M.; Echenique, P. M.; Saito, M.; Hasegawa, S. Role of Spin-Orbit Coupling and Hybridization Effects in the Electronic Structure of Ultrathin Bi Films. *Phys Rev Lett* **2006**, *97* (14), 146803. https://doi.org/10.1103/PhysRevLett.97.146803.

(6) Toudert, J.; Serna, R.; Camps, I.; Wojcik, J.; Mascher, P.; Rebollar, E.; Ezquerra, T. A. Unveiling the Far Infrared-to-Ultraviolet Optical Properties of Bismuth for Applications in Plasmonics and Nanophotonics. *Journal of Physical Chemistry C* **2017**, *121* (6), 3511–3521. https://doi.org/10.1021/acs.jpcc.6b10331.

(7) Palik, E. D. Handbook of Optical Constants of Solids. *Handbook of Optical Constants of Solids* **2012**, *1*, 1–804. https://doi.org/10.1016/C2009-0-20920-2.

(8) Cheng, Z.; Milne, T.; Salter, P.; Kim, J. S.; Humphrey, S.; Booth, M.; Bhaskaran, H. Antimony Thin Films Demonstrate Programmable Optical Nonlinearity. *Sci Adv* **2021**, *7* (1). https://doi.org/10.1126/SCIADV.ABD7097.

(9) Soria, E.; Gomez-Rodriguez, P.; Tromas, C.; Camelio, S.; Babonneau, D.; Serna, R.; Gonzalo, J.; Toudert,. Self-Assembled, 10 Nm-Tailored, Near Infrared Plasmonic Metasurface Acting as Broadband Omnidirectional Polarizing Mirror. *Adv Opt Mater* **2020**, *8* (21), 2000321. https://doi.org/10.1002/ADOM.202000321.
